# CD200-CD200R imbalance correlates with microglia and pro-inflammatory activation in rat spinal cords exposed to amniotic fluid in retinoic acid-induced spina bifida

**DOI:** 10.1038/s41598-018-28829-5

**Published:** 2018-07-13

**Authors:** Marc Oria, Rebeca L. Figueira, Federico Scorletti, Lourenco Sbragia, Kathryn Owens, Zhen Li, Bedika Pathak, Maria U. Corona, Mario Marotta, Jose L. Encinas, Jose L. Peiro

**Affiliations:** 1 Center for Fetal and Placental Research, Division of Pediatric General and Thoracic Surgery, Cincinnati Children’s Hospital Medical Center (CCHMC), Cincinnati, OH USA; 2 Laboratory of Experimental Fetal Surgery “Michael Harrison”, Division of Pediatric Surgery, Department of Surgery and Anatomy, Ribeirao Preto Medical School, University of Sao Paulo-USP, Ribeirao Preto, Brazil; 3 Department of Pediatric Surgery, La Paz University Hospital, Madrid, Spain

## Abstract

Spina bifida aperta is a congenital malformation characterized by the failure of neural tube closure resulting in an unprotected fetal spinal cord. The spinal cord then undergoes progressive damage, likely due to chemical and mechanical factors related to exposure to the intrauterine environment. Astrogliosis in exposed spinal cords has been described in animal models of spina bifida during embryonic life but its relationship with neuroinflammatory processes are completely unknown. Using a retinoic acid-induced rat model of spina bifida we demonstrated that, when exposed to amniotic fluid, fetal spinal cords showed progressive astrogliosis with neuronal loss at mid-gestation (E15) compared to unexposed spinal cords. The number of microglial cells with a reactive phenotype and activation marker expression increased during gestation and exhibited progressive disruption in the inhibitory immune ligand-receptor system. Specifically we demonstrate down-regulation of CD200 expression and up-regulation of CD200R. Exposed spinal cords demonstrated neuroinflammation with increased tissue water content and cytokine production by the end of gestation (E20), which correlated with active Caspase3 expression in the exposed layers. Our findings provide new evidence that microglia activation, including the disruption of the endogenous inhibitory system (CD200-CD200R), may participate in the pathogenesis of spina bifida through late gestation.

## Introduction

Neural tube defects, the most prevalent and disabling of all the congenital malformations, occur with an incidence of 1 per 1,000 births^1^. These serious malformations of the central nervous system (CNS) develop when the canal of the brain or spinal cord remains persistently open to the environment. This process during gastrulation, occurs during the fourth week of human gestation, and the lack of closure result in anencephaly and/or spina bifida. These developmental defects cause severe brain impairment or local disruption of vertebrae and spinal axonal pathways. The unprotected neural tissues of the developing fetus suffers progressive damage due to exposure to various chemical and mechanical factors in the intrauterine environment^2^. As a result, the neurological consequences at birth are irreversible and sometimes devastating^2,3^.

Neuroinflammation and microglia activation are prominent features in neurodegenerative disease and important mechanisms in chronic neurodegeneration after spinal cord injury^4^. Neuronal death and astrocyte proliferation with reactive astrogliosis are well described in this scenario^5,6^ but the mechanisms that control gliosis and neuroinflammation in spina bifida remain unclear.

Involvement of the immune ligand-receptor CD200-CD200R complex has been demonstrated in the regulation of cytokine production^7^. The CD200 transmembrane glycoprotein, mostly expressed in neurons, interacts with its receptor, CD200R which is expressed in the CNS almost exclusively in microglia as well as other CNS macrophages, to inhibit microglial priming and holds microglia in a quiescent state^8^. During normal development, this cell-cell communication reinforces an anti-inflammatory response, and is responsible for microglial development and renewal in the developing CNS^8^.

However, disruption of the CD200-CD200R signaling can cause microglial over reactivity^9–12^. In spina bifida the loss of neural tissue^2,13^ can disrupt the CD200-CD200R ratio, thus leading to over activation of the microglia, induction of the neuroinflammatory process, and neurodegeneration^14,15^. Knowing the exhisting neural tissue damage in utero in spina bifida and given the importance of this immune ligand-receptor system in regulating microglial expression during brain development and microglial priming; we hypothezised that spina bifida suffer a progressive astrroglial and microglial activation during gestation and the microglia priming may be related to the alteration of the CD200-CD200R system. This study examines progression during gestation of astrogliosis and neuroinflammation analyzing the effect of CD200-CD200R on microglia reactivity during gestation in the rat fetal model of retinoic acid-induced spina bifida aperta.

## Material and Methods

All experimental protocols were approved by the Institutional Animal Care and Use Committee at the Children’s Hospital of Cincinnati, and followed guidelines set forth in the National Institutes of Health Guide for Care and Use of Laboratory Animals (IACUC 2013-0293).

### Retinoic acid (RA)-induced spina bifida animal model

36 timed-pregnant Sprague-Dawley rats weighing 200–250 g (Charles River Laboratories, Inc, Wilmington, MA) were housed individually at 22°C on a standard dark:light schedule (12:12 hours,i.e., light 7:00–19:00) with ad libitum access to water and standard chow. Mating date was defined as E-1 and plug date as 0. Trans retinoic acid (RA) (Sigma-Aldrich Chemical, St. Louis, MO) solubilized at room temperature in olive oil was used within 1 h of preparation and protected from light. On E10 at 10 am, 20 timed-pregnant rats were gavaged with RA 100 mg/kg in olive oil to induce spina bifida in the litter and 16 timed-pregnant rats were gavaged with olive oil as a sham.

### Tissue Processing

Litters from timed-pregnant rats were harvested at three timepoint in gestation (E15, E17, E20). Tissue samples were collected and included spinal cords exposed and unexposed to amniotic fluid in spina bifida fetuses and spinal cords from sham fetuses. For gene and protein expression, spinal cords were removed, immediately snap frozen, and stored at −80 °C until use. Specimens for histological analysis were isolated and fixed in 4% paraformaldehyde for 24 hours, processed, and embedded in paraffin. For flow cytometry and water content experiments, fresh cords were extracted and processed according to specific protocols described below.

### RNA Extraction

Tissues were homogenized (IkaT10 basic Ultra-Turrax homogenizer, USA) in RLT buffer and RNA extracted using an RNeasy Plus Mini Kit (Qiagen Science, Hilden, Germany) following manufacturer’s protocol. Samples were quantified by spectrophotometry (Epoch Biotek, Biotek Instruments, Winooski, Vermont). A 1-µg RNA/sample was reverse transcribed into cDNA using the RT^2^ First Strand Kit (Qiagen Sciences, city, Maryland, USA). A 1-µg cDNA sample was used to set up RT-qPCR using TaqMan^R^ gene expression assay (Applied Biosystems, Foster City, CA, USA) (Supplementary Table 1) and 7500 Fast Real-Time PCR System. Target genes were normalized using HPRT1 as endogenous control; their relative quantification of transcript expression was performed using the 2^−Δ ΔCt^ method (C_t_ represents the threshold cycle). Samples were run in triplicate.

### Protein extraction

Total protein extracted from spinal cord samples was 20–30 mg. Tissue samples were homogenized by sonication (Fisher Scientific, Pittsburgh, PA) in N-PER (Thermo Fisher Scientific, Rockford, IL) + Proteinase K + PhosphoSTOP (Roche Diagnostics GmbH, Mannheim, Germany) in 1.5-mL tubes, and centrifuged at 10,000 rpm for 5 minutes. Protein concentration was assessed using the BCA-mini method (Thermo Fisher Scientific, Rockford, IL).

### Western Blot

Samples (total 20 µg protein) were loaded into 4–12% acrylamide gels, electrophoresed for 50 minutes at 200 V, and transferred to polyvinylidene difluoride (PVDF) membranes (Life Technologies, Eugene, OR) for 1.5 hour at 20 V. After blocking with bovine serum albumin (BSA) (5%) for 1 hour, membranes were hybridized with antibodies anti-NeuN (Abcam, Cambridge, MA) (1:1000), anti- GFAP (DAKO, Carpinteria, CA) (1:1000), anti-Iba1 (Wako, Richmond, VA), anti-GAPDH (Abcam) (1:10000), and anti-βActin (Abcam) (1:10000) overnight at 4 °C. Membranes were washed, hybridized with secondary antibody (Abcam) (1:10000) for 1 hour, and developed using a SuperSignal West Pico Chemiluminescent substrate (Thermo Fisher Scientific, Rockford, IL). Bands were quantified using Image J software and normalized to βActin expression.

### Immunofluorescence

Sections were dried and permeabilized with 0.1% Triton X-100 (Sigma Aldrich, St. Louis, MO) in phosphate buffered saline (PBS), blocked fornon-specific binding for 1 hour with 5% BSA in PBS, and then incubated with primary antibody βIII-Tub (Covance, San Diego, CA) (1:1000), anti-NeuN (Chemicon, Temula, CA), (1:500), anti-GFAP (DAKO) (1:1000), anti-Iba1 (Wako) (1:1000), MHCII (Thermo Fisher Scientific) (1:100), Ki76 (Thermo Fisher Scientific) (1:500) overnight at 4 °C in a humid chamber. Sections were washed and incubated for 1 hour with Alexa Fluor 488, Alexa Fluor 568, or Alexa Fluor 647 conjugated secondary antibodies (Life Technologies, Eugene, OR) (1:1000) in the dark at room temperature. Slides were washed, mounted with DAPI mounting media (SouthernBiotech, Birminghan, AL), and visualized with a Nikon fluorescent microscope (Nikon Inc., Melville, NY).

### Lumimex Methods

Cytokine (IL1β, IL6, and INFγ) concentrations in the sample homogenate were determined by enzyme-linked immunosorbent assay (ELISA) using MilliplexTM Multiplex kits (MilliporeSigma, Darmstadt, Germany) according to manufacturer’s protocol. Briefly, in duplicate in a 96-well black plate, a 25-μL sample was incubated with 25-μL antibody-coated beads overnight at 4 °C on a plate shaker. Plates were then washed twice using the BioTek 405 TS (BioTek, Winooski, VT), 25 μL of secondary antibody was added, and samples were incubated at room temperature for 1 hour with shaking. Finally, 25 μL of strept-avidin-RPE was added directly to the secondary antibody and incubated for 30 minutes at room temperature with shaking. After plates were washed 2 more times, 150 μL of sheath fluid was added. Plates were shaken for 5 minutes and then read using luminex technology on the Milliplex Analyzer (MilliporeSigma, Darmstadt, Germany). Concentrations were calculated from standard curves using recombinant proteins and expressed in pg/ml.

### Wet/dry measurements

Harvested pieces from spinal cords (exposed and nonexposed) from 6 spina bifida fetuses were extracted, weighed (Sartorius Scale, Gottingen, Germany), and dried to a constant weight in an oven (Precision Scientific, Chicago, IL) at 60 °C for 24 h. Percentage water and water content of each sample were calculated as follows^16^:

$$\begin{array}{c}{\rm{ \% }}\,{\rm{w}}{\rm{a}}{\rm{t}}{\rm{e}}{\rm{r}}\,{\rm{c}}{\rm{o}}{\rm{n}}{\rm{t}}{\rm{e}}{\rm{n}}{\rm{t}}=100\,({\rm{w}}{\rm{e}}{\rm{t}}\,{\rm{w}}{\rm{e}}{\rm{i}}{\rm{g}}{\rm{h}}{\rm{t}}-{\rm{d}}{\rm{r}}{\rm{y}}\,{\rm{w}}{\rm{e}}{\rm{i}}{\rm{g}}{\rm{h}}{\rm{t}})/{\rm{w}}{\rm{e}}{\rm{t}}\,{\rm{w}}{\rm{e}}{\rm{i}}{\rm{g}}{\rm{h}}{\rm{t}}\\ {\rm{a}}{\rm{n}}{\rm{d}}\,\\ {\rm{w}}{\rm{a}}{\rm{t}}{\rm{e}}{\rm{r}}\,{\rm{c}}{\rm{o}}{\rm{n}}{\rm{t}}{\rm{e}}{\rm{n}}{\rm{t}}\,=\,({\rm{w}}{\rm{e}}{\rm{t}}\,{\rm{w}}{\rm{e}}{\rm{i}}{\rm{g}}{\rm{h}}{\rm{t}}-{\rm{d}}{\rm{r}}{\rm{y}}\,{\rm{w}}{\rm{e}}{\rm{i}}{\rm{g}}{\rm{h}}{\rm{t}})/{\rm{d}}{\rm{r}}{\rm{y}}\,{\rm{w}}{\rm{e}}{\rm{i}}{\rm{g}}{\rm{h}}{\rm{t}}\end{array}$$

### Flow cytometry

The flow cytometry protocol for immune cells used as previously described^17^. Samples for flow cytometry analysis were dissected and processed following Miltenyi Biotec protocol for neural tissue dissociation kit (Miltenyi Biotec, Auburn, CA, USA). Briefly, spinal cords from spina bifida fetuses exposed and nonexposed and sham animals were harvested and digested with papain enzyme at 37 °C in rotation using gentle MACS Dissociator (Miltenyi Biotec). Cell suspension was meshed with 70-µm Strainer (BD Pharmingen, San Jose, CA) and labeled at 4 °C for 30 min with a specific antibodies panel previously reported^17^ for flow cytometry. Cell populations were analyzed by flow cytometry on a LSRII Flow Cytometer system (BD Pharmingen) depending the immunolabeling for microglia/macrophages (CD45/CD11b/c^+^), and activated microglia (CD200R^+^ and MHCII^+^). Antibodies used for flow cytometry experiment were: Fixable viability staining-APC-eFluor780 (eBioscience, Rockford, IL), CD45-PECy7 (Biolegend, San Diego, CA), CD11b/c-PerCP-Cy5.5 (Biolegend), CD200R-FITC (Biolegend), MHCII-PE (eBioscience). Data were analyzed using FlowJo software (v.10).

### Statistical analysis

Categorical results were expressed as proportions and percentages; continuous results were means ± standard error (SE), Error bars are standard errors calculated upon averaging ΔCt values within sample groups and transformed to fold change errors with error propagation for the gene relative expression (2−ΔΔCt) and means ± standard deviation (SD) for protein expression for RA-treated and time-matched controls. For Comparison between groups ANOVA test was performed. In comparison of not normal distributed groups, differences between means were compared using the Mann-Whitney U test. The Graph Pad Prism 7 (GraphPad Software Inc., La Jolla, CA) package was used for figures and statistical calculations. *A p* value* < *0.05 was considered statistically significant.

### Ethics approval and consent to participate

All experimental protocols were approved by the Institutional Animal Care and Use Committee at Cincinnati Children’s Hospital Medical Center and followed guidelines set forth in the National Institutes of Health Guide for Care and Use of Laboratory Animals (IACUC 2013–0293), with all methods and experiments performed in accordance with the relevant guidelines and regulations.

### Data availability

Data supporting the findings of this study are available within the article and its supplementary information.

## Results

**Figure 1:**
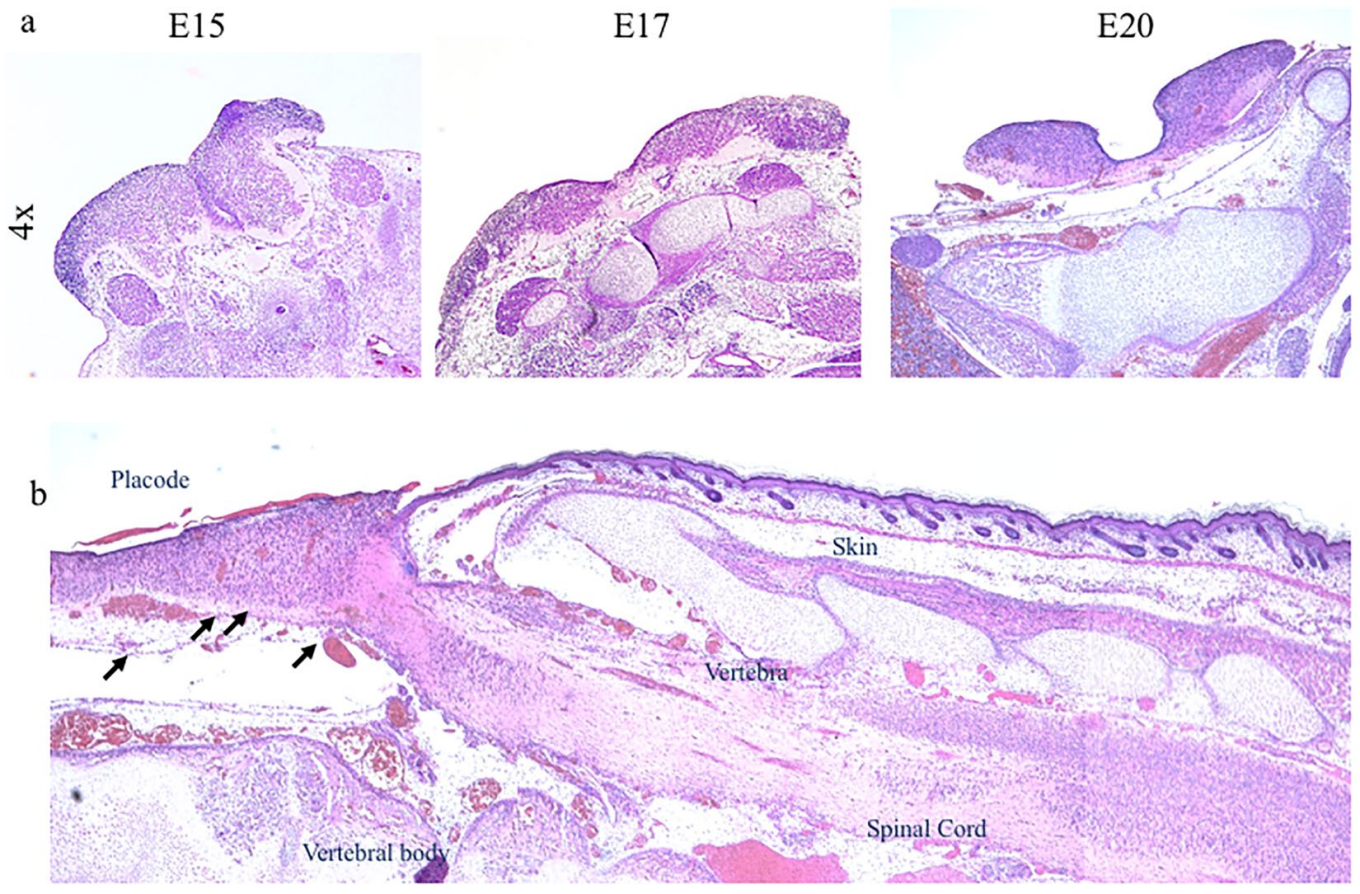
RA-induced spina bifida aperta in rat fetuses during gestation showing spinal cord sections by hematoxylin and eosin (H & E) stain. (**a**) Exposed cord at gestational days E15, E17, and E20 (4×). (**b**) At E20 (sagittal view), exposed cord shows altered cellular density and blood vessel extravasation (arrows) (4×).

In our rat model, retinoic acid at 100 mg/kg on gestational day E10 induced spina bifida in 71% of the fetuses. No fetuses developed spina bifida in the 16 sham animals. Litter size was significantly smaller in the RA-induced model compared to sham, 8.6 ± 4.2 and 13.9 ± 2.1, respectively (*p < 0.05) (Supplementary Fig. 1). Fetuses at E15, E17, and E20 with open RA-induced spina bifida showed classic defects that included open posterior arc, dysraphic spinal cord (i.e., lacking dura), and exposure to the ammonitic fluid environment. Variations were observed in the shape of the open spinal cord and the extent of neural tissue loss. Spina bifida exposed cords showed progressive tissue loss throughout gestation (Fig. 1).

### Effects of amniotic fluid exposure

**Figure 2:**
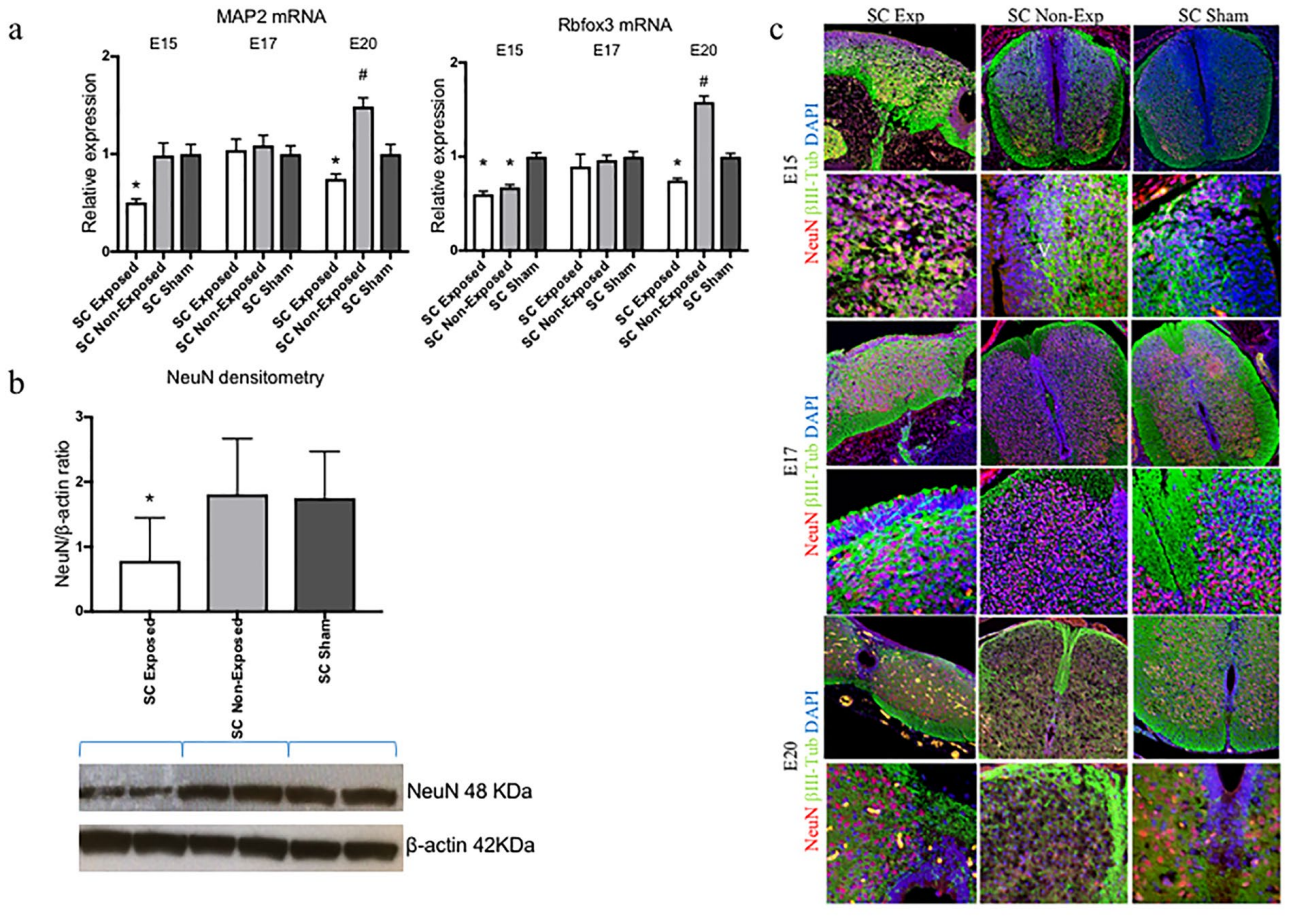
Neuronal expression. (**a**) MAP2 and Rbfox3 gene expression in exposed and unexposed spina bifida cords, and sham spinal cords (SC). Values are means ± SE of relative expression at E15, E17 and E20 for RA-treated (6 fetuses/group) and time-matched controls (6 fetuses/group) (^#^and *p < 0.05). (**b**) Western Blot confirmed that NeuN protein expression was reduced at E20 (*p < 0.05) in spina bifida spinal cords exposed to the amniotic fluid but remained normal in unexposed spina bifida and sham cords. Data (mean ± SD, 6 fetuses/group) of the densitometry ratio and representative cropped bands from the same gel NeuN (48KDa) and β-actin (42 KDa). Relative expression of NeuN normalized to β-actin from spinal cord lysates (mean ± SD 6 fetuses/group). (**c**) βIII-Tub (green), Neun (red), and DAPI (blue) immunostaning at E15, E17 and E20 for exposed and unexposed spinal cords in spina (10×, 40×).

After RA exposure and non-closure of the posterior arc, the neural tissues remained exposed to the amniotic fluid. Compared with sham controls at E15 and E20, gene expression of the neural microtubule-associated protein 2 (MAP2) and RNA binding protein Fox-1 Homolog 3 (Rbfox3) were downregulated in the exposed spinal cord but significantly increased in the unexposed spinal cord at E20 (Fig. 2a). NeuN expression was significantly (*p < 0.05) lower in exposed spinal cords than either unexposed or sham cords (Fig. 2b) at E 20. βIII-Tub and NeuN staining identified unprotected neurons exposed to the amniotic environment, which caused neural loss and resulted in abnormal cytoarchitecture of the exposed spinal cord (Fig. 2c). This architecture was also affected in regions where reduced cellular density was clearly distinguished with extravasation of the blood vessels (arrows) (Fig. 1b).

### Astrogliosis in the injured spinal cord

**Figure 3:**
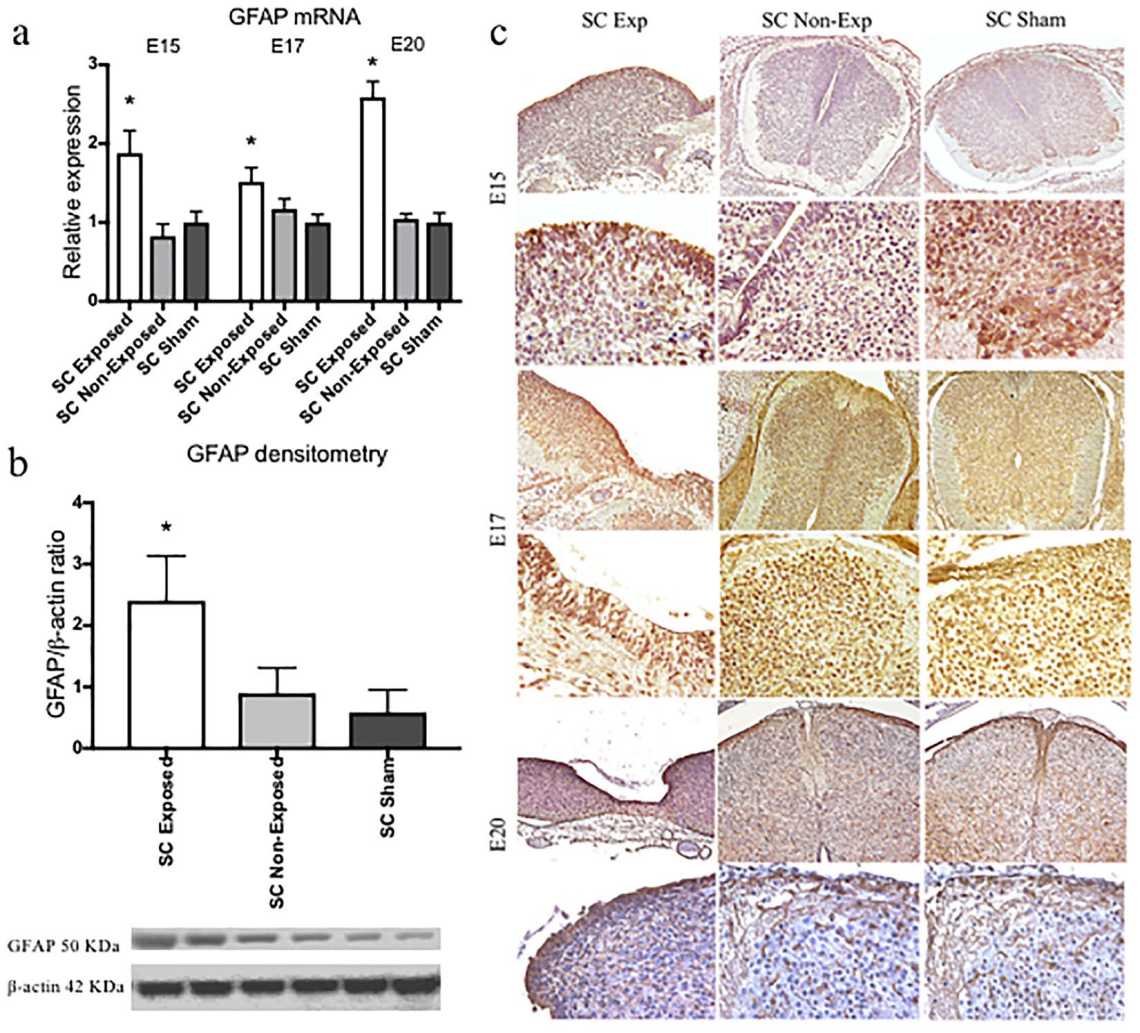
Immunohistochemistry staining and quantification of GFAP expression. (**a**) GFAP relative expression increased in spina bifida in exposed neural tissue compared with unexposed spina bifida and sham animals. Values (means ± SE, 6 fetuses/group) of relative expression (2^−ΔΔCt^) for RA-treated and time-matched controls (*p < 0.05). (**b**) Western blot confirmed GFAP protein levels were higher in exposed spinal cords in spina bifida than unexposed or sham cords (*p < 0.05). Densitometry ratio (mean ± SD, 6 fetuses/group) and representative cropped bands from the same gel GFAP (50KDa) and β-actin (42 KDa). (**c**) GFAP-immunoreactive astrocytes in the spinal cord sections of spina bifida exposed, unexposed, and sham fetuses at E15, E17, and E20 (10× and 40×).

**Figure 4:**
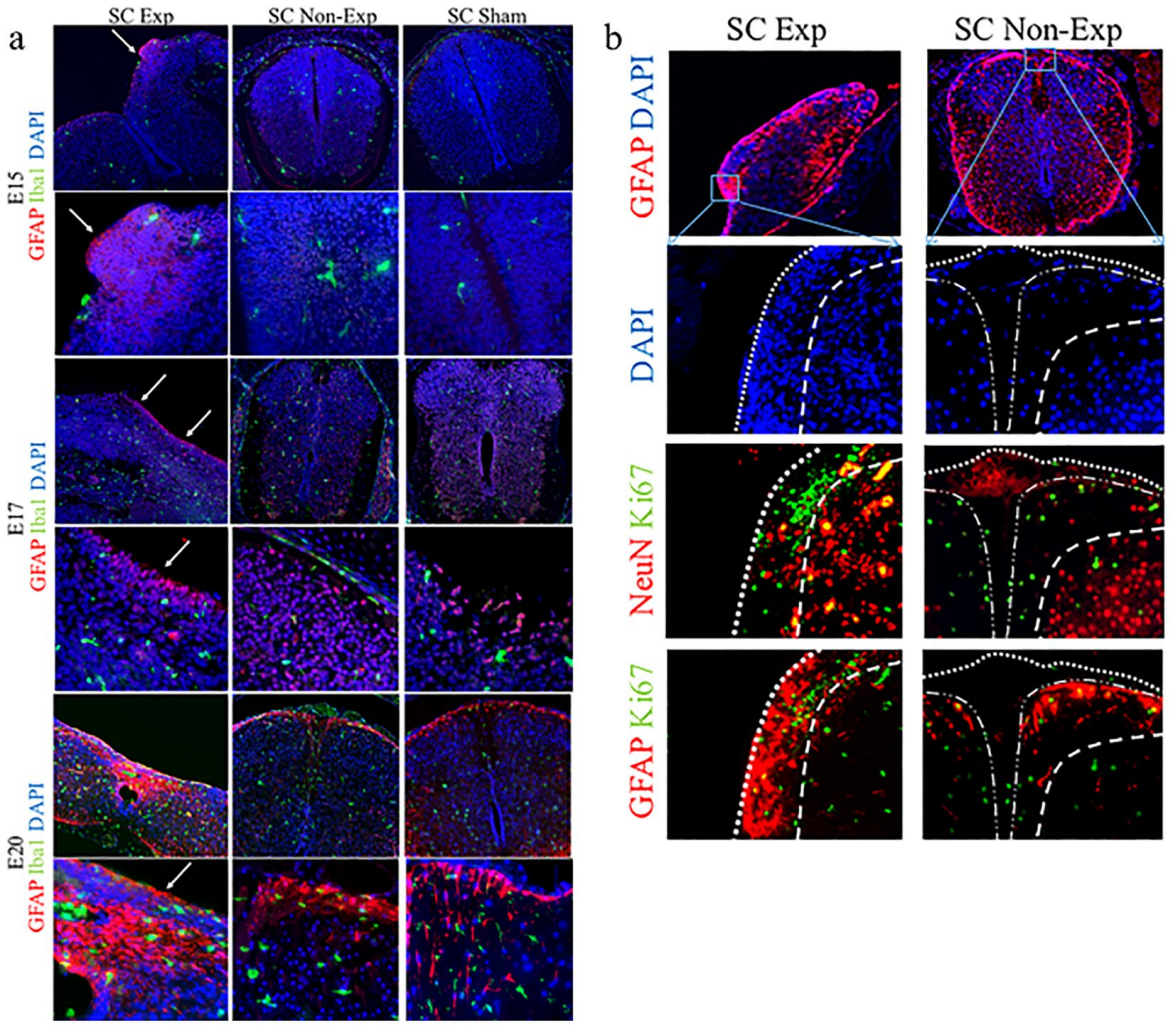
Progressive immunoreactive astrocytes in exposed tissue in spina bifida. (**a**) Exposed and unexposed spinal cords from spina bifida fetuses at E20. Progressive GFAP (red) immunoreactive astroctyte cells at E15, E17, and E20 in spina bifida exposed spinal cords (arrows). Reactive microglia stained with Iba1 (green) and DAPI (blue). (**b**) GFAP-immunoreactive GFAP (red) and DAPI (blue) coronal section (10×). Immunoreactive and proliferative (green) astrocytes in exposed layers in aggregates (40×). No patterned neural distribution in exposed spinal cords in spina bifida fetuses stained with NeuN (red)(40×). Proliferative astrocytes GFAP (red) colocalized with Ki67 (green) in spinal cords exposed in spina bifida fetuses at E20.

At each of the timepoints, GFAP expression was dramatically higher in the exposed spinal cords than either the unexposed region of cord from the same spina bifida fetus or sham cords, especially at E20 (*p* < 0.05) (Fig. 3a); this was confirmed by Western blot analysis (Fig. 3b). Distribution of GFAP-positive astrocytes appeared as numerous aggregates throughout the lesion near the exposed neural tissue in spina bifida fetuses, but followed normal distribution in the spinal cords for unexposed and sham at E20 (Fig. 3c). GFAP expression showed a progressive pattern through gestation from E15 to E20. At E15, high expression of GFAP was detected in some areas in the exposed cords but not distributed equally in the exposed layers compared with incipient low expression in the non-exposed and sham cords (Fig. 4a). Higher expression of GFAP in exposed layers also correlated with higher levels of proliferation identified by immunofluorescence for Ki67 (Fig. 4b).

### Neuroinflammation and reactive microgliosis in exposed neural spinal cord

**Figure 5:**
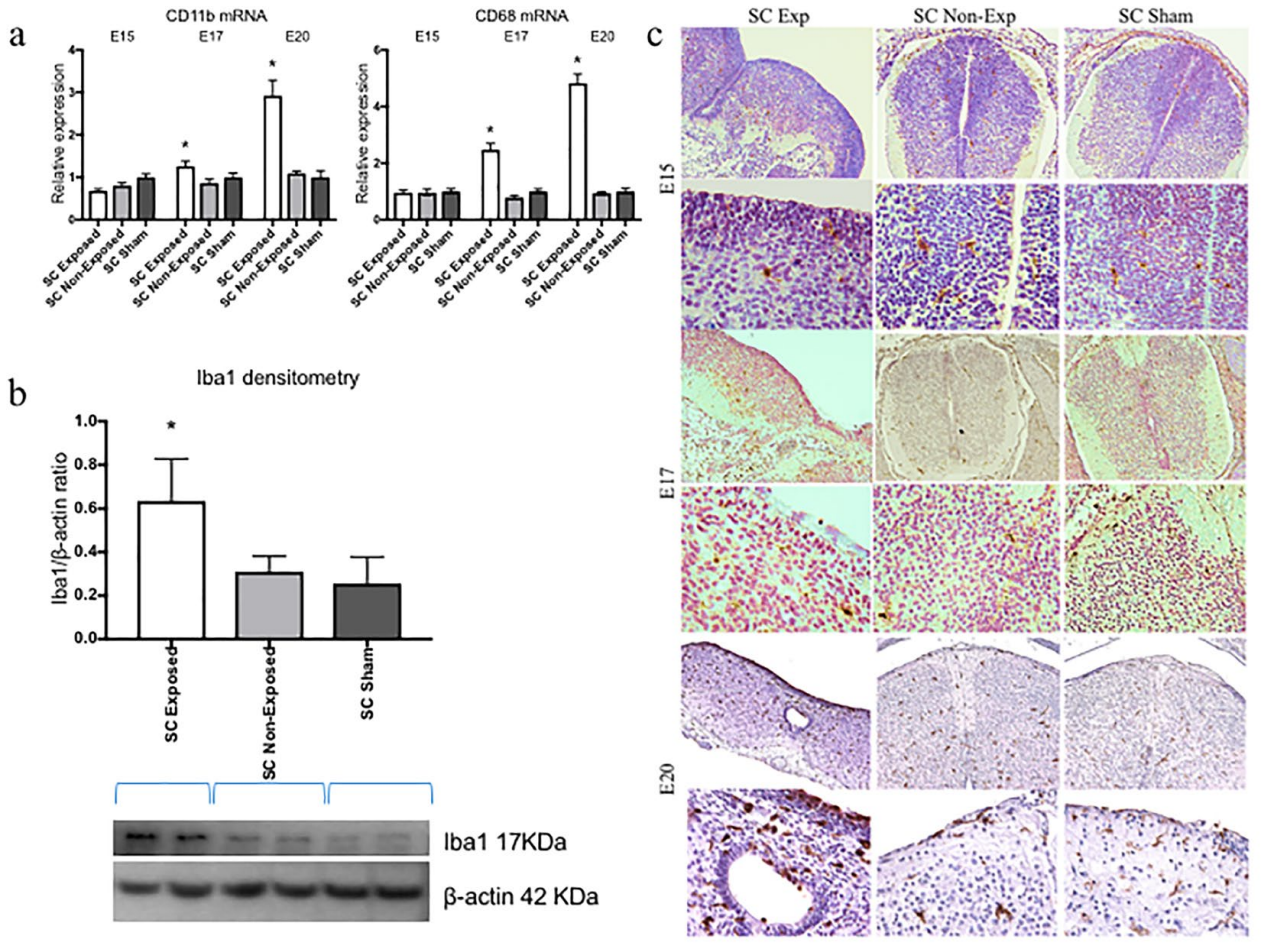
Immunofluorescence staining and quantification of microglia expression. (**a**) Comparison at E15, E17, and E20 for microglial markers CD11b and CD68 relative gene expression up-regulation in neural tissues of exposed and unexposed spina bifida and sham spinal cords. Values are means ± SE (6 fetuses/group) of relative mRNA expression (2^−ΔΔCt^) for RA-treated and the time-matched controls (*p < 0.05) at E20 (n = 6). (**b**) Western blot results confirmed Iba1 protein expression increased in the exposed spinal cord for spina bifida compared with unexposed or sham spinal cords at E20 (*p < 0.05). Data presented as means ± SD (6 fetuses/group) of the densitometry ratio and representative cropped bands from the same gel Iba1 (17KDa) and β-actin (42 KDa). (**c**) Representative images of Iba1-immunoreactive microglial cells in spinal cord sections (10×, 40×) at E15, E17 and E20 for exposed and unexposed spina bifida and sham spinal cords.

**Figure 6:**
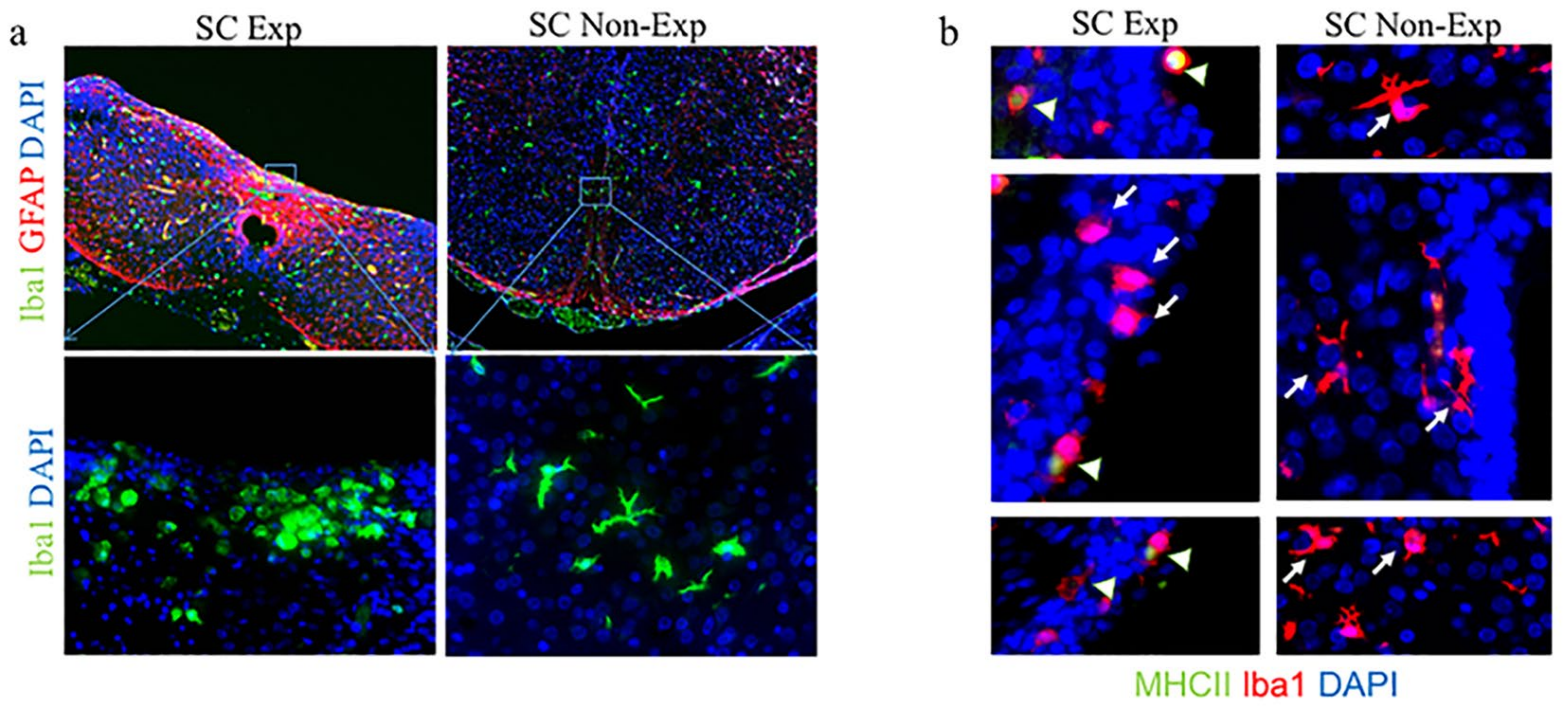
Progressive microglia activation in spinal cord exposed tissue in spina bifida. (**a**) Immunoreactive astrocytes GFAP (red), microglia Iba1 (green), and DAPI (blue) in exposed and un-exposed spina bifida spinal cords; top panel (magnification 20×). Activated rounded microglial morphology in exposed spinal cord in the layers exposed to the amniotic fluid compared with resting morphology in the unexposed spinal cord in spina bifida pups; lower panel (magnification 40×). (**b**) Iba1 (red), MHCII (green) and DAPI (blue) immunostaining in exposed and unexposed spina bifida cords. Iba1+/MHCII− (arrows) and Iba1+/MHCII+ (arrowheads) microglial cells in layers of the spinal cord exposed to the amniotic fluid (magnification 100×) also showed activated phenotype compared with Iba1+/MHCII−(arrows) microglial cells in resting phenotype with extended projections in unexposed spinal cords (magnification 100×).

To confirm the microglia activation in spina bifida fetuses, we assessed morphological changes and quantified microglial-specific markers Iba1 and CD11b (both constitutive markers). CD11b and CD68 gene expression was progressively significantly up-regulated after E17 of gestation in spinal cords exposed to the amniotic fluid compared with unexposed spinal bifida or sham spinal cords (p < 0.05) (Fig. 5a). In Western Blot analyses, Iba1 protein expression was higher in the spinal cords exposed to amniotic fluid than those unexposed at E20 (*p* < 0.05) (Fig. 5b). Furthermore, of the microglia present, an increase in the population with activated morphology was detected by immnohistochemistry at E17 and E20 but not at E15 (Fig. 5c). More Iba1 cells with activated morphology were seen at E20 showing the progressive increase in microglial cells (Fig. 6a). Mixed populations of microglial cells were also present in the spina bifida exposed spinal cord, including Iba1 + and MHCII + cells, and Iba1 + cells MHCII- cells; this finding thus differentiated between activated and unactivated cells. In contrast, only Iba1 + MHCII- cells were observed in unexposed spinal cord as resting phenotype with extended projections (Fig. 6b).

### CD200-CD200R alteration correlates with cytokine production in spina bifida

**Figure 7:**
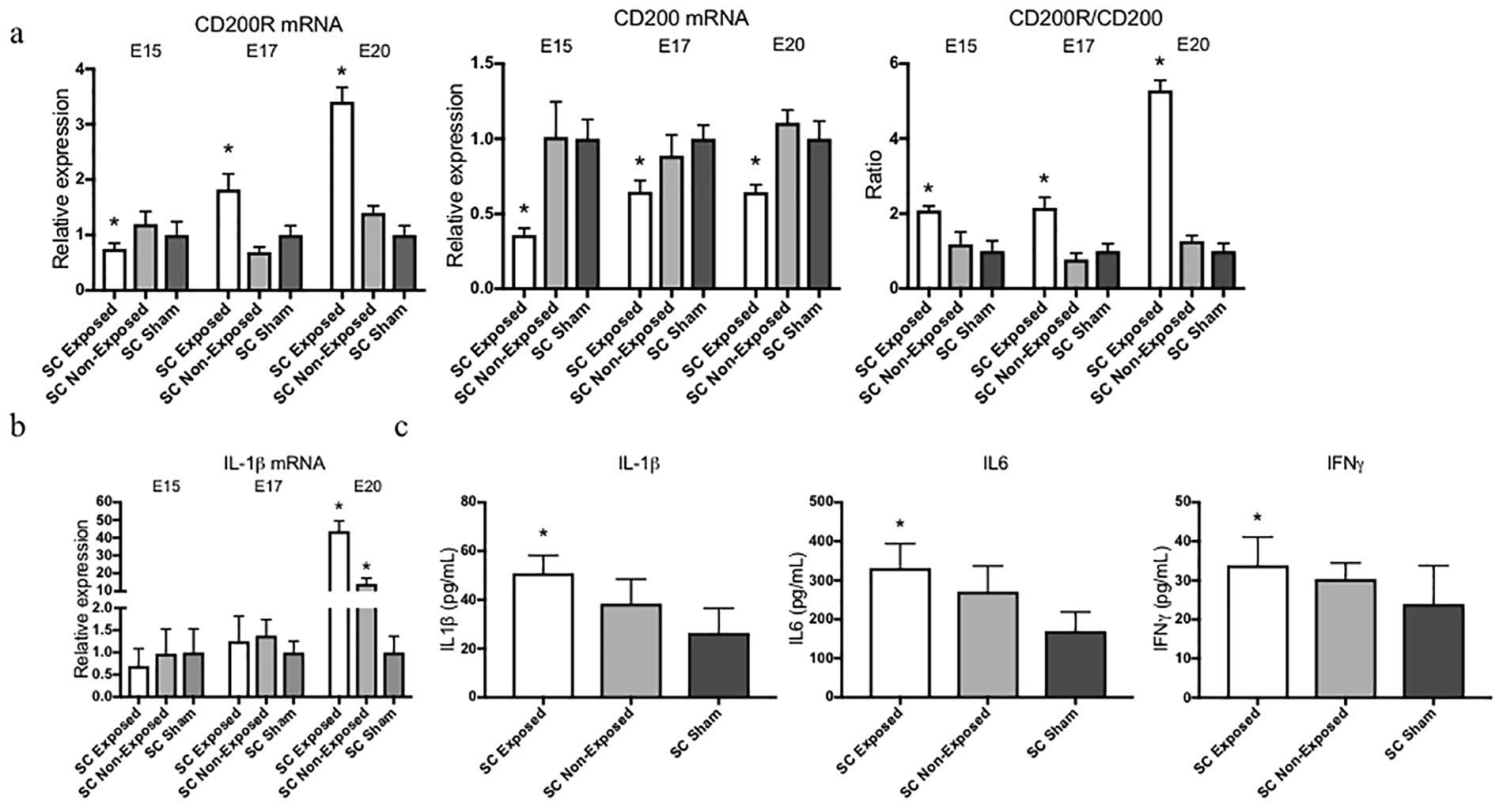
Progressive disruption of immune ligand-receptor (CD200-CD200R) in spina bifida aperta. (**a**) Comparison of CD200R, CD200, and CD200/CD200R ratio relative expression in exposed neural tissue, unexposed, and sham animals at different gestational age (E15, E17 and E20). Means ± SE (n = 6 fetuses/group) of relative expression (2^−^ΔΔCt) for RA-treated and time-matched controls; (*p < 0.05). (**b**) IL1β gene relative expression confirmed the upregulation and correlation between microglial activation, neuroinflammation, and cytokine production in exposed spina bifida spinal cord at E20 of gestation. (**c**) Cytokine (IL1β, IL6, INF γ) protein expression by Luminex exhibit higher cytokine expression in the exposed spinal cord of spina bifida compared with unexposed or sham rats (n = 5 fetuses/group) at E20. Values are means ± SD (*p < 0.05).

The relationship between the imbalance of CD200-CD200R and the pro-inflammatory cytokines in spina bifida was demonstrated by the expression of the ligand CD200, mostly by neurons, and the expression of receptor CD200R, mostly by microglia. Compared with unexposed and sham spinal cords, exposed neural tissue showed reduced CD200 expression, increased CD200R expression, and disruption of the normal CD200R-CD200 ratio throughout gestation with more significance at E20 (*p* < 0.05) (Fig. 7a). In the exposed spina bifida spinal cords, this activation correlated with pro-inflammatory cytokine production as demonstrated by the gene expression of IL1β at E20 and by the increase of IL1β, IL6 and IFNγ protein expression at E20 (*p* < 0.05) (Fig. 7b,c).

### Flow cytometry evaluation of activated microglia/macropahges cell populations

**Figure 8:**
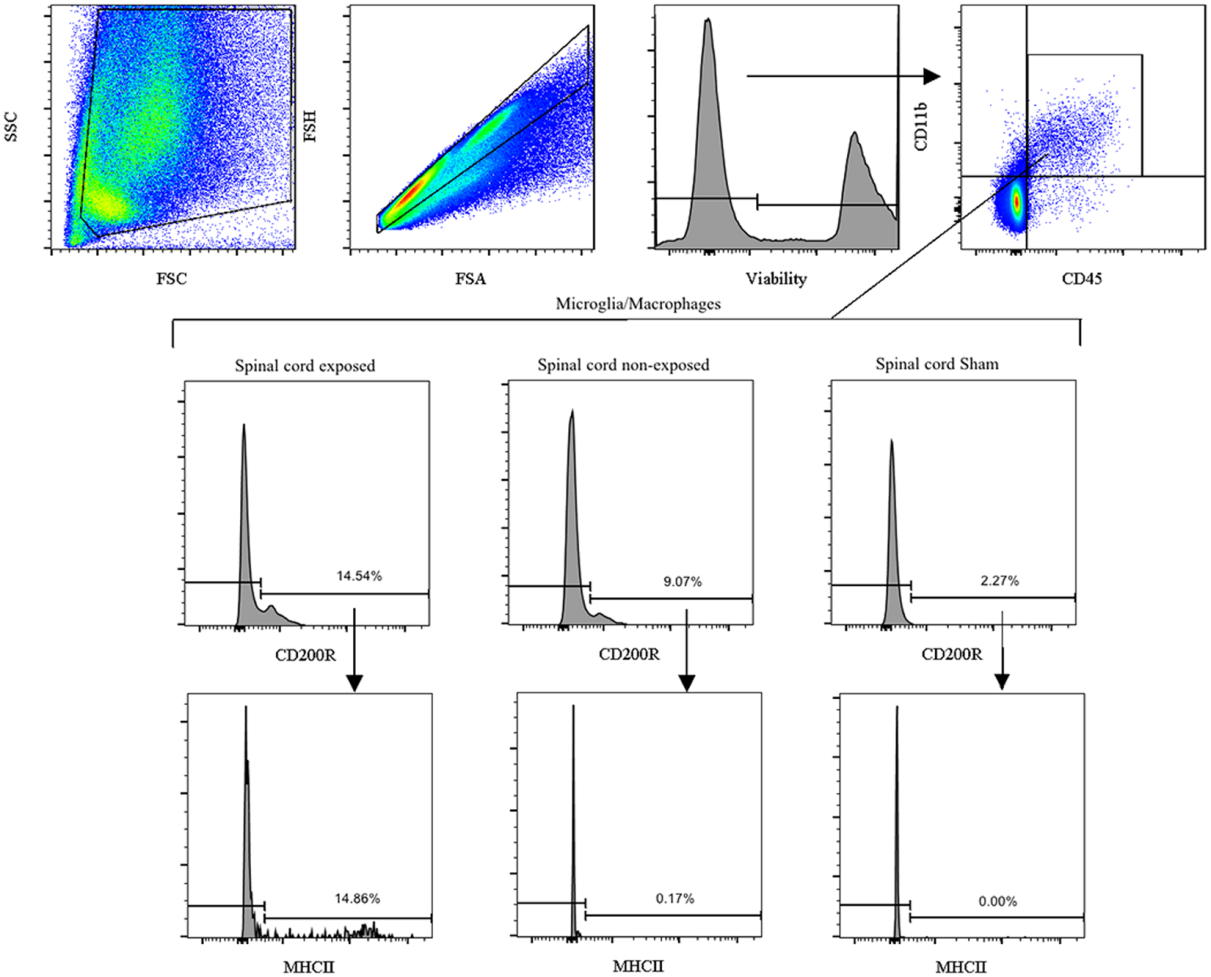
Flow cytometry analysis of cell microglia/macrophages populations in spinal cord of spina bifida rats at E20. Single cell suspension from spina bifida exposed, unexposed, and sham spinal cords for activated microglial/macrophage cell population analysis. Cells were first selected based on viability and CD45 staining. Microglia/macrophages cell population were selected for CD45 and CD11b. Activated microglia/macrophages were selected for CD200R^+^ and then for MHCII^+^. SSC = Side scatter, FSC = Forward Scatter.

Cells were initially sorted on the basis of viability and the presence of CD45 staining. Microglia/Macrophages cell population were selected for CD45 and then positively selected for CD11b/c. Within populations of CD45/CD11b/c cells on cords in spina bifida and sham spinal cords, exposed spinal cords in spina bifida fetuses had increased CD200R^+^ cells (14.54%) compared with unexposed spina bifida and sham, respectively (9.07% and 2.27%). Within the CD200R^+^ positive subpopulation, MHCII^+^ was also increased in exposed cords (14.86%) compared with unexposed cords in spina bifida (0.17%) and sham (0.00%) (*p* < 0.05) (Fig. 8). Fetuses they comprised

### Water Content

In assessing water content and percentage by dry and wet methods, respectively, significant differences were found in the cord tissues of the same spina bifida pup with exposure to the amniotic environment (Supplementary Fig. 2a). Water content was significantly higher in exposed than unexposed spinal cords in the same spina bifida fetuses. A fairly small change in percentage water content actually reflected large changes in tissue water content (Supplementary Fig. 2b) (*p < 0.05, paired Student’s T-test).

### Spina bifida exposed spinal cord induced apoptosis

**Figure 9:**
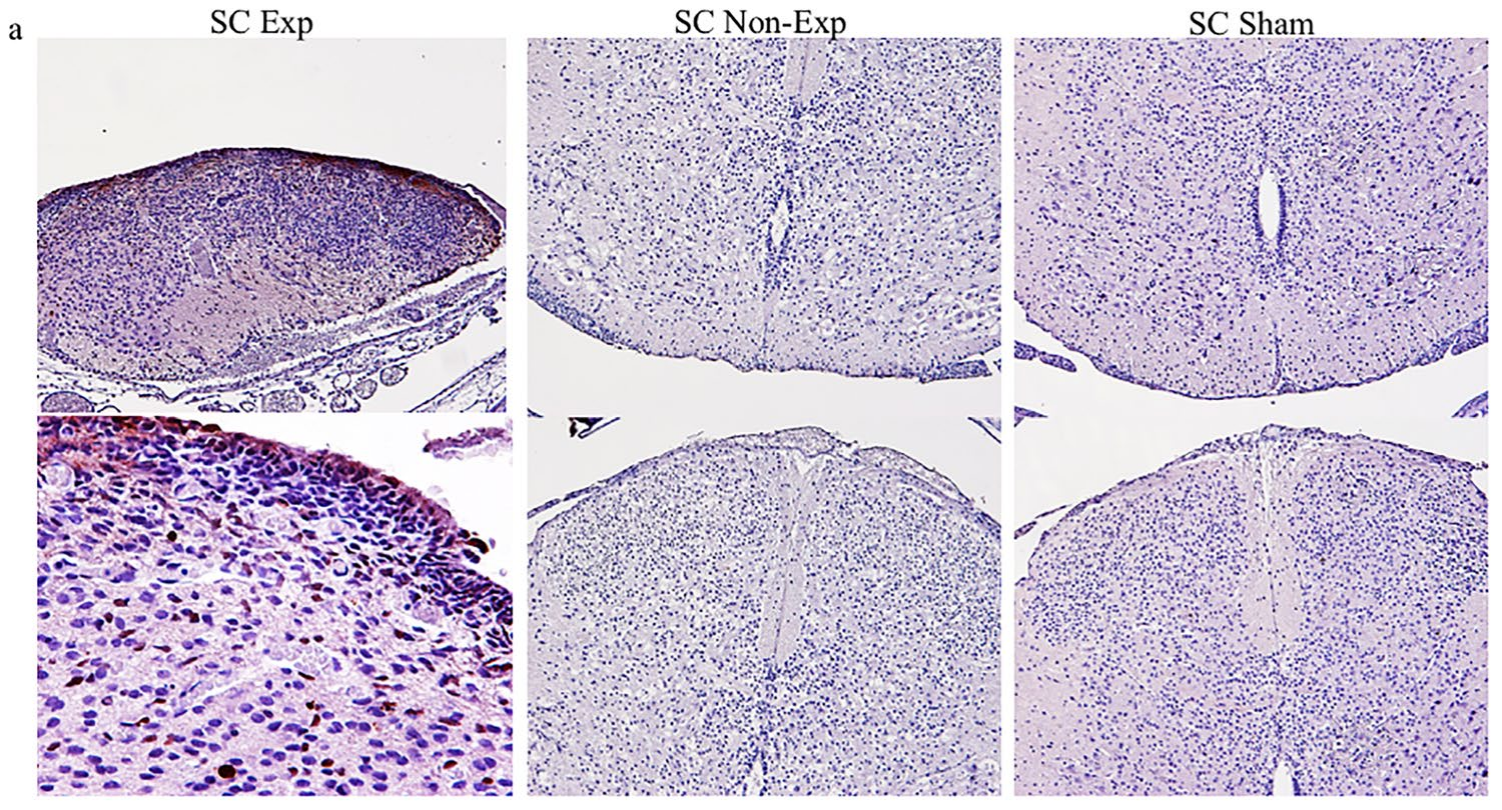
Cleaved-caspase 3 expression in exposed spinal cord (sc) in spina bifida aperta at E20. Cleaved-caspase 3 immunoreactive cells in the spinal cord sections (top row 20× and bottom row 40×). Increased expression seen in exposed spinal cord compared with no expression in unexposed spina bifida and sham spinal cords at E20.

To test if spinal cord exposure to amniotic fluid induced apoptosis by activation of the neuroinflammatory process, tissue sections were stained for cleaved caspase 3. In spina bifida fetuses, spinal cords exposed to the amniotic fluid exhibited a greater density of cleaved-caspase-3 + cells in the exposed layers at E20 of gestation. No positive cells were found in the unexposed spina bifida spinal cords and sham controls (Fig. 9).

## Discussion

Our study describes the disruption of normal prenatal CNS evolution with presence of neuroinflammatory processes and microglia activation in an RA-induced rat model of spina bifida. One of the possible mechanism involved in priming microglia is the immune suppressor ligand-receptor CD200-CD200R. The imbalance on CD200-CD200R is related to the activation of microglia and pro-inflammatory cytokines. Our findings provide evidence for the first-time into the presence of the microglia with activated phenotype in the pathophysiology of spina bifida.

Our characterization of neuroinflammation in a retinoic-acid induced animal model of neural tube defects builds upon other *in vivo* studies that have profiled lesions in spina bifida^18^. By performing analysis at the gestation time points (E15, E17, and E20) we were able to identify progressive effects of exposure of the spinal cord to amniotic fluid. Firstly, pathophysiological changes along the spinal cord by analysis of neural cell types (i.e., astrocytes, neurons, microglia) could be compared between exposed and unexposed spinal cords in the same spina bifida fetuses. The progression of the astrogliosis and neural loss in the exposed spinal cords started very early in gestation at E15 compared with the normal development and differentiation at E18 and after birth. Second, understanding the braking effect of the CD200-CD200R interaction resulted; specifically, microglia maintained in the quiescent state led to abnormal activation in the injured tissue just before birth, especially at E20, inducing a pathological neurinflammatory response.

In spina bifida the fetal spinal cord is exposed to the enzymatic action of amniotic fluid^3^. Chemical and physical erosion result in loss of neural tissue, both aggravating the primary lesion and changing the cytoarchitecture of the spinal cord^13^.

In histological samples, regions of reduced cellular density were also observed; importantly, these regions, which contained edema and extravasation of the blood vessels, were clearly identifiable in the sagittal view along the spinal cord. The increased water content in exposed Vs. unexposed neural tissues in the same animal may reflect a process that occurs with spinal cord injuries in adults^19^. For example, when tissues in the blood-brain-barrier are broken, extravasation of the vessels, activation of the inflammatory and apoptotic processes increase the water content in injured neural tissue.

In this RA-induced rat model, the most common neuropathological feature described was the presence of reactive astroglia in spinal cords of fetuses with spina bifida exposed to the amniotic fluid. Astrogliosis has been described before in this model^8^ and other spina bifida animal models^20,21^, but in our model we detected earlier activation at E15 in the exposed spinal cords from spina bifida fetuses.

Astrocytes, which are the neuromodulatory and neurotrophic elements in the CNS, respond to any stress or tissue damage by proliferation, hypertrophy, and migration. Given the strong association of glial fibrillary acidic protein (GFAP) with astrocytic cell proliferation and differentiation^22,23^, assessment of its expression may elucidate the pathophysiology of the neural tissue in open neural tube defects. The GFAP is up-regulated in reactive astrocytes^24,25^

Astrogliosis has been previously described in animal models of spina bifida^18,20^ at E16.5-E18. Normal astrogenesis in rat fetuses starts at E18 and continues after birth until post-natal day7^26^. However, mechanisms that control gliosis in spina bifida remain unclear. This reactive astrogliosis, which is not only a marker of neuropathology, also plays a role in the response to the injury by regulating neuroinflammation and reparation^17,25,27–30^. Our data suggest the formation of a gliosis in injured tissues in spina bifida rat fetuses. Specifically, the reactive astrocytes proliferate in the exposed layers trying to protect the neural tissue from the hostile environment from mid-gestation (E15). Microglial cells migrate, mix with the astrocytes in the exposed layers in the spina bifida fetuses, and thus create the inflammatory response to the injury.

Microglia, the resident macrophage population of the CNS, maintain homeostasis for normal function during fetal development; their involvement includes removal of apoptotic cells and debris^31^, synaptic pruning^32^, modulation of the synaptic transmission^33^, and angiogenesis^34^. Responding quickly after injury or stress, they offer protective effects. However, if unrestricted, these effects can be deleterious leading to neural degeneration in the injured tissues. In prenatal cases, disruption of normal CNS maturation by neuroinflammation and microglia activation can cause serious cognitive and bahavioral defects^35–38^. These mechanisms, which may play an important role in spinal bifida, have not been investigated for this pathology. Microglial cells are the sentinel cells derived from the primitive yolk sac; these cells migrate and invade the CNS during early gestation E8-E8.5^36^ and are the macrophages resident in the CNS when lack of closure is induced in retinoic acid models (E10). Here, using other microglial activation markers (e.g., CD11b, MHCII, CD68), we demonstrated that microglial cells are activated in the exposed spinal cord in spina bifida fetuses. More importantly, this microgliosis and neuroinflammatory response is progressive during gestation. As shown by a previously described flow cytometry strategy^17^, we corroborated the increase of activated microglia/macrophages in exposed neural tissues in the spina bifida fetuses at day 20 of gestation.

### Disruption of the inhibitory ligand-receptor system, CD200-CD200R

Our findings provide new evidence that microglia activation may participate in the etiology and pathogenesis of spina bifida, especially towards the end of gestation. One mechanism implicated in microglial activation in the lesion was the disruption of the inhibitory ligand-receptor system (CD200-CD200R). Many studies suggest that CD200-CD200R signaling down-regulates the activity of myeloid cells and that microglia derived from myeloid progenitor cells migrated at an early stage from the yolk sac^39^. In analyzing the expression of CD200-CD200R, we demonstrated that disruption of this inhibitory immune ligand-receptor system in the lesion led to microglial activation at E20. Downregulation of CD200, probably due to loss of neural tissue by erosion and cell death caused by amniotic fluid exposure. Activation of astrocytes and microglia in the damaged tissue upregulated the expression of CD200R^27,40^. These dysregulations disrupted the balance of the immune ligand:receptor ratio (CD200-CD200R)^41,42^. The disruption then exacerbated microglial activation which in turn increased MHCII expression and pro-inflammatory cytokine (IL1β, IL6, and IFNγ)^10–12^ production that was detected in the exposed spinal cords at E20.

### Study limitations

It is unlikely that CD200-CD200R system is solely responsible for priming microglia, especially at early gestational stages when the neural tube defect occurs, as CD200 and CD200R are not expressed at that time. Therefore, additional experiments are needed to elucidate the role and mechanism of macrophage/microglia in the injured spinal cord at earlier gestational ages.

## Conclusion

Neural tube defects, including spina bifida aperta, are the most common permanent disabling birth defects. Despite fetal surgery to repair or cover the spinal cord at mid gestation, significant anomalies already established persist throughout pregnancy and beyond birth. The physiopathology of the complex inflammatory activation remains poorly understood. Characterization of CNS alterations in the open lesion of RA-induced spina bifida in rats in this study provides evidence for the first-time of a relationship between reactive astrocytes, neural tissue loss, and neuroinflammation in Spine Bifida. Furthermore, we have demonstrated that activation of the neuroinflammatory mechanisms and primed microgliosis involves the disruption of the endogenous inhibitory system (CD200-CD200R). Chronic microglial activation is an important component of neurodegenerative diseases, and this chronic neuroinflammatory component likely contributes to injury in spina bifida aperta after birth.

## Electronic supplementary material

Supplementary Information
