# Supplementary material for: CD200-CD200R imbalance correlates with microglia and pro-inflammatory activation in rat spinal cords exposed to amniotic fluid in retinoic acid-induced spina bifida
Source: Sci Rep. 2018 Jul 13;8:10638. doi: 10.1038/s41598-018-28829-5 (PMC6045622; doi:10.1038/s41598-018-28829-5)
Supplement: Supplementary file 1 — Supplementary Information [file 41598_2018_28829_MOESM1_ESM.pdf]

CD200-CD200R imbalance correlates with microglia and pro-inflammatory activation in rat spinal cords exposed to amniotic fluid in retinoic acid-induced spina bifida

Marc Oria, Rebeca L Figueira, Federico Scorletti, Lourenco Sbragia, Kathryn Owens, Zhen Li, Bedika Pathak, Maria U Corona, Mario Marotta, Jose L Encinas, Jose L Peiro

Supplementary Figure 1

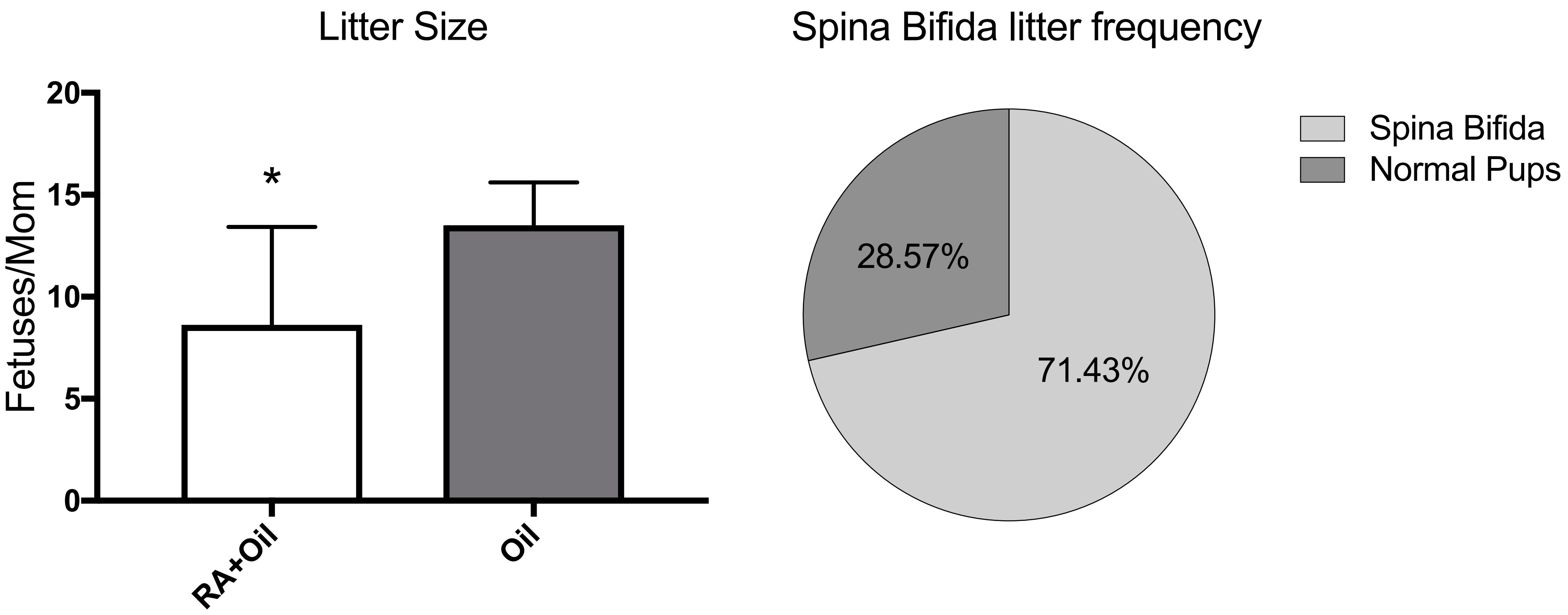

Supplementary Figure 2

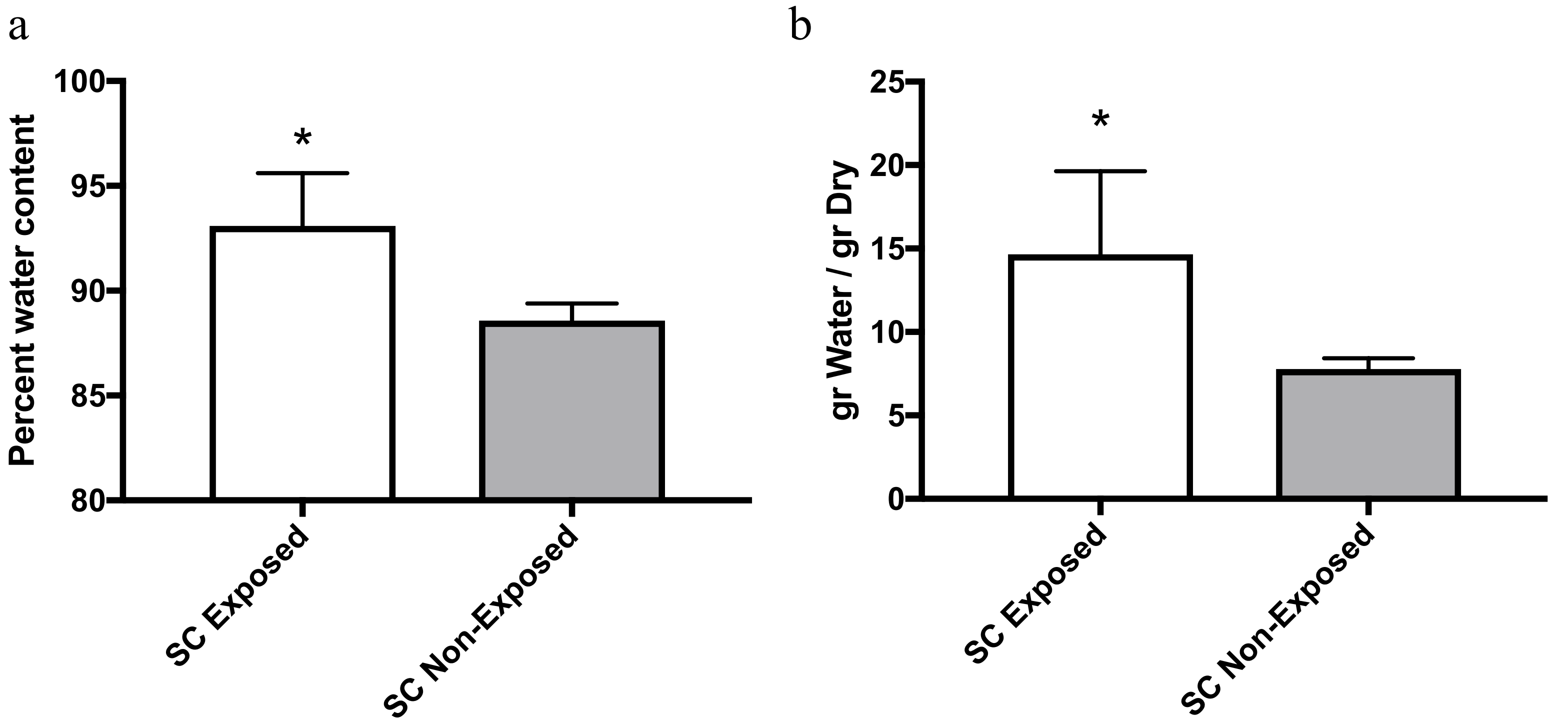

Supplementary Table 1

mRNA	Name	*Assay code number
Itgam	Integrin alpha M, CD11b	Rn00709342_m1
Cd200r1	CD200 receptor 1	Rn00576646_m1
Cd200	CD200 molecule	Rn01646320_m1
Cd68	CD68 molecule	Rn01495634_g1
Il1b	Interleukin 1 beta	Rn00580432_m1
Hprt1	Hypoxanthine phosphoribosyltransferase 1	Rn01527840_m1
Map2	Microtubule associated protein 2	Rn01401429_m1
GFAP	Glial fibrillary acid protein	Rn01253033_m1

Table1. TaqMan probes for gene expression assay

*Probes code assay from Applied Biosystems, Foster City, CA, USA
